# Advances in endogenous hypochlorous acid-mediated regulation of tumor cell fates

**DOI:** 10.3389/fcell.2026.1822165

**Published:** 2026-05-28

**Authors:** Weikang Zhao, Xiaoling Cui, Kexin Zheng, Fengying Du, Yanxu Li, Baoshan Cai, Liang Shang, Changqing Jing, Leping Li

**Affiliations:** 1 Department of Gastrointestinal Surgery, Shandong Provincial Hospital Affiliated to Shandong First Medical University, Jinan, China; 2 Shandong Provincial Laboratory of Translational Medicine Engineering for Digestive Tumors, Shandong Provincial Hospital, Jinan, China; 3 Medical Science and Technology Innovation Center, Shandong First Medical University and Shandong Academy of Medical Sciences, Jinan, China; 4 Department of Gastrointestinal Surgery, Shandong Provincial Hospital, Shandong University, Jinan, China

**Keywords:** apoptosis, cancer therapy, hypochlorous acid, myeloperoxidase, oxidative stress, reactive oxygen species

## Abstract

Hypochlorous acid (HOCl), an important reactive oxygen species generated by myeloperoxidase (MPO), is widely involved in the pathogenesis of various conditions, including cardiovascular, renal, and neurodegenerative diseases, as well as cancer. This article reviews recent progress regarding the roles of HOCl in determining cell fate and driving disease progression. We first introduce the mechanisms of HOCl formation and its explicit mechanistic links to these pathologies. Subsequently, we summarize the oxidative modification of biological macromolecules, focusing specifically on proteins. Furthermore, we highlight the regulatory functions of HOCl in cell fate decisions, its disruptive interactions with subcellular organelles, and its paradoxical effects within the tumor microenvironment. Finally, we discuss potential clinical therapeutics and diagnostics that target HOCl-associated signaling pathways. Ultimately, this review aims to provide a systematic understanding of HOCl in disease development and to stimulate the design of novel therapeutic strategies.

## 1 Introduction

Reactive oxygen species (ROS) play complex and critical roles in cellular processes, encompassing cell signaling, innate immune defense, and pathological pathways. Among these, hypochlorous acid (HOCl) is particularly significant as a potent oxidizing agent. Within living organisms, HOCl serves an essential function in innate immunity; however, its dysregulated production directly drives the pathogenesis of numerous chronic inflammatory conditions (such as atherosclerosis, rheumatoid arthritis, and neurodegenerative disorders) and cancer. Rather than merely exerting broad cytotoxicity, this physiological imbalance actively precipitates severe cellular damage through specific molecular pathways.

Recent research reveals that HOCl functions not only as an inflammatory byproduct and mediator of tissue damage but also as a crucial signaling molecule. By oxidatively modifying biomacromolecules—particularly targeting specific amino acid residues in proteins—HOCl profoundly influences critical cell fate decisions, including apoptosis, proliferation, senescence, and differentiation. Moreover, HOCl profoundly disrupts subcellular organelle function. In the context of oncology, HOCl exerts paradoxical effects. It can promote tumorigenesis by inhibiting apoptosis and facilitating tumor cell survival; conversely, it can also selectively induce apoptosis in malignant cells, thereby inhibiting tumor progression. The diverse biological roles of HOCl and its significant impact on disease pathogenesis underscore its potential as a targeted therapeutic vulnerability. This concept tightly aligns with the broader therapeutic framework of redox adaptation, where the inherently dysregulated redox signaling and altered antioxidant defenses of cancer cells are exploited to selectively induce cytotoxicity via ROS amplification—a strategy already demonstrating substantial promise with H_2_O_2_-generating agents (Ali et al., 2024). Consequently, various HOCl-targeting strategies are currently under development, including diagnostic probes for early tumor detection, novel anticancer therapeutics, and topical formulations for dermatological malignancies.

## 2 Introduction to hypochlorous acid

Peroxidases, a class of oxidoreductases widely distributed in living organisms, catalyze the oxidation of various substrates using hydrogen peroxide (H_2_O_2_) or other peroxides. Among these enzymes, heme peroxidases—including myeloperoxidase (MPO), eosinophil peroxidase (EPO), lactoperoxidase (LPO), and thyroid peroxidase (TPO)—utilize H_2_O_2_ to oxidize halide or pseudohalide ions, generating the corresponding hypohalous acids. Neutrophil-derived MPO is crucial for bacterial eradication, whereas EPO primarily targets invading parasites. LPO, a potent antimicrobial enzyme present in secretions such as saliva, tears, and breast milk, is essential for clearing respiratory pathogens (Singh et al., 2024).

**FIGURE 1:**
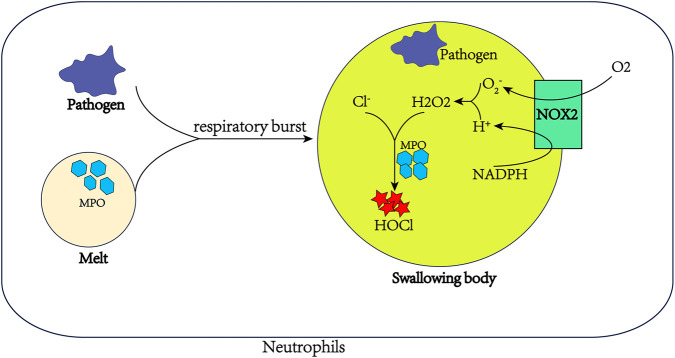
HOCl Generation in the Phagolysosome. HOCl is generated in phagolysosomes when MPO catalyzes the reaction of Cl^−^ and H_2_O_2_, which is produced by the NOX2/NADPH pathway during the neutrophil respiratory burst upon pathogen invasion.

Myeloperoxidase (MPO), which constitutes approximately 5% of neutrophil azurophilic granule proteins, is also localized in the lysosomes of polymorphonuclear leukocytes and macrophages (Thekkan et al., 2019; Liu T. W. et al., 2021). Utilizing a novel near-infrared fluorescent probe (FNIR-HOCl) developed specifically for lysosomal HOCl detection, researchers have confirmed that phagocyte lysosomes serve as the primary site for HOCl synthesis (Jiang et al., 2023). Upon encountering pathogens, phagocytes fuse with lysosomes to form phagolysosomes, triggering a respiratory burst. During this process, membrane-bound NADPH oxidase (NOX2) oxidizes NADPH to generate protons while reducing oxygen to superoxide. The resulting hydrogen peroxide is then converted by MPO, in the presence of chloride ions, into a bactericidal burst of HOCl (Anees et al., 2021; Droste et al., 2021; Mahorivska et al., 2025) (Figure 1). In macrophages, the gradual acidification of the phagosome culminates in a rapid release of HOCl, indicating that phagosome-lysosome fusion is essential for providing the chloride ions necessary to sustain MPO activity (Thekkan et al., 2019).

Direct quantification of HOCl in human fluids remains technically challenging and is not common in clinical settings due to its extreme reactivity and instability (Jeelani et al., 2020). Nevertheless, during the respiratory burst, activated neutrophils utilize up to 80% of the produced H_2_O_2_ to synthesize HOCl. The local concentration of HOCl at inflammatory sites can reach millimolar levels, with production rates ranging from 20 to 400 μM/h (Misztal et al., 2022). Extensive clinical evidence links elevated plasma levels of MPO and its oxidative byproducts (e.g., HOCl) to inflammatory pathologies such as atherosclerosis, demonstrating a clear correlation between plasma MPO levels and cardiovascular disease risk (Hu et al., 2021). Consequently, excessive HOCl underpins the pathogenesis of multiple disorders. For instance, in rheumatoid arthritis (RA), abundant synovial neutrophils secrete MPO to generate high levels of HOCl, which erodes cartilage—especially glycosaminoglycans—thereby accelerating joint destruction (Leopold and Schiller, 2024). Furthermore, elevated MPO and HOCl levels are strongly associated with renal, neurodegenerative, and pulmonary diseases, while significantly impacting tumorigenesis (Lin et al., 2024). These pathological relationships have been further validated in MPO-knockout murine models, including studies on cerebral ischemic injury (Coulibaly et al., 2021) and non-small cell lung cancer (Valadez-Cosmes et al., 2023).

## 3 Types of hypochlorous acid-modified amino acid residues

As a highly reactive oxidizing agent, hypochlorous acid (HOCl) disrupts cellular functions and induces cell death by rapidly altering the structures of DNA, RNA, and proteins (Baruah et al., 2023). Phospholipids, constituting the bulk of cellular membranes, represent early key targets. In oxidatively damaged tissues, the accumulation of lysophosphatidylcholine (LPC) (Prabutzki et al., 2024) correlates strongly with atherosclerosis (Cao et al., 2024) and valvular disease (Sylvester et al., 2023). LPC is primarily generated through two pathways: enzymatic cleavage of phosphatidylcholine by phospholipase A2, and the direct reaction of lipids with reactive oxygen species like HOCl (Prabutzki et al., 2024). These HOCl-oxidized lipids exhibit significant cytotoxicity toward endothelial cells (HUVEC-ST), triggering mitochondrial depolarization, apoptosis, G1/S cell-cycle arrest, and the activation of redox-sensitive p38 kinase (Robaszkiewicz et al., 2014). Furthermore, morphological analyses indicate that HOCl induces DNA strand breaks and mutagenic lesions; unrepaired damage prior to replication can initiate tumorigenesis or perpetuate inflammatory disorders (Tantry et al., 2018).

Among these diverse biomolecules, proteins represent the primary targets for direct HOCl-mediated oxidative damage. HOCl oxidizes amino acid side chains, fragments polypeptides, and promotes aberrant cross-linking and aggregation, ultimately dictating protein fate and downstream cellular responses (Hawkins, 2020). Given their abundance and critical roles in executing genetic information (Zhang and Wang, 2023), maintaining protein architecture is essential; disruption at any structural level can precipitate disease. Critically, even when the global protein conformation remains intact, covalent alterations to amino acid side chains—analogous to traditional post-translational modifications (PTMs) such as methylation, acetylation, or phosphorylation (Lee et al., 2023) —can profoundly alter protein activity. Unlike H_2_O_2_, which possesses low direct reactivity towards most biomolecules and largely depends on metal-catalyzed Fenton chemistry to generate highly toxic hydroxyl radicals (Ali et al., 2024), HOCl exhibits a much broader reactivity profile. It readily reacts with glycine (Gly), serine (Ser), alanine (Ala), valine (Val), arginine (Arg), lysine (Lys), histidine (His), cystine (Cys dimer), glutamine (Gln), and asparagine (Asn), alongside Tyr, Cys, and Met (Andrés C. M. et al., 2022). Overall, sulfur-containing residues exhibit the strongest reactivity with HOCl, following a general reactivity hierarchy: Met > Cys > disulfide bonds > His > Trp > Lys > Tyr > Arg > Gln > Asn (Andrés C. M. C. et al., 2022).

**FIGURE 2:**
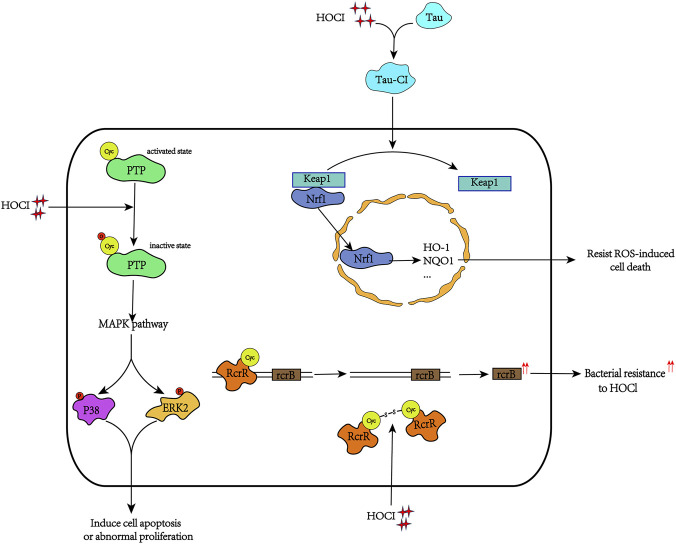
Types of Hypochlorous Acid-Modified Amino Acid Residues. HOCl modifies various amino acid residues, regulating the Keap1-Nrf2 and RcrR pathways to mediate cellular antioxidant defense and bacterial HOCl resistance, and modulates cell fate via the MAPK pathway.

Taurine, a highly abundant free amino acid in the brain, reacts with neutrophil-derived HOCl to form taurochloramine (Tau-Cl). Tau-Cl subsequently oxidizes the thiol group of Keap1, disrupting the Keap1-Nrf2 complex. The liberated Nrf2 translocates to the nucleus and binds to the antioxidant response element (ARE), upregulating antioxidant enzymes (HO-1, NQO1) and glutathione biosynthetic enzymes (GCLC, GCLM). This cascade enhances the cellular capacity to scavenge ROS, conferring resistance against H_2_O_2_-induced apoptosis (Seol et al., 2024). Conversely, HOCl-mediated oxidation of methionine exclusively yields methionine sulfoxide (Nascimento et al., 2022), while the oxidation of cysteine or cystine proceeds via RS-Cl intermediates to generate sulfinic or sulfonic acids (Hawkins, 2020). HOCl frequently induces reversible (e.g., sulfenic acid, disulfide bond) or irreversible (e.g., sulfinate, sulfonate) thiol modifications. Reversible cysteine modifications carry significant structural and functional consequences; remarkably, many bacteria exploit these modifications to bolster their adaptive defense mechanisms (Crompton et al., 2023). For example, HOCl induces the formation of disulfide-linked oligomers between Cys54 and Cys95 of the HOCl-sensitive transcriptional repressor *RcrR*. This structural shift causes *RcrR* to dissociate from its promoter region (*PrcrA*), upregulating the expression of *rcrA*, *rcrR*, and *rcrB*, thereby enhancing bacterial HOCl resistance (Sultana et al., 2022) (Figure 2).

Beyond sulfur-containing residues, HOCl chlorinates the indole ring of tryptophan (Trp), generating 3-chloroindolenine, which forms a previously unidentified cyclic indole amide. In this reaction, Trp cross-links with the backbone nitrogen atom of an adjacent glycine, creating an aromatic six-membered ring—a structural modification known to compromise matrix metalloproteinase (MMP) activity and contribute to related pathologies (Fu et al., 2004). Furthermore, HOCl-mediated oxidation of tyrosine leads to either the formation of dityrosine (driving protein cross-linking) (Dalle-Donne et al., 2001) or direct chlorination into 3-chlorotyrosine (3-ClTyr)—the gold-standard biomarker for MPO-mediated chlorination activity *in vivo* (Di Salvo et al., 2023). A recent metabolomics analysis of saliva from patients with neurodegenerative diseases revealed that 3-ClTyr levels correlate strongly with Alzheimer’s disease (AD) progression and severity (François et al., 2024). Finally, HOCl reacts with the side chains of lysine and histidine to form N-chloramines. These chloramines retain the oxidative capacity of HOCl and can target other amino acid residues, facilitating intramolecular chlorine transfer and generating diverse oxidation products (Hallberg et al., 2022; Wang et al., 2022). Notably, while lysine and arginine serve as secondary targets (Andrés C. M. C. et al., 2022), lysine chloramines can act as Cl^+^ donors to facilitate tyrosine chlorination within specific α-helical Y-x-x-K motifs (Choe et al., 2015). These diverse amino acid modifications, particularly the chlorination and oxidation of tyrosine and cysteine residues, lead to the formation of Advanced Oxidation Protein Products (AOPPs), which serve as critical mediators of inflammation (Garibaldi et al., 2017).

## 4 Regulation of cell fate by hypochlorous acid

### 4.1 HOCl induces and suppresses apoptosis

Exposure of cultured cells to pathological concentrations of hypochlorous acid (HOCl) triggers apoptosis through overlapping pathways, predominantly involving mitochondrial injury, the secondary generation of reactive oxygen species (ROS; e.g., hydroxyl radicals and singlet oxygen), and caspase-3 activation (Jeelani et al., 2020). In the intrinsic apoptotic pathway, cellular stress induces outer mitochondrial membrane permeabilization, facilitating the release of cytochrome c into the cytosol. In mammalian cells, upon binding to cytochrome c, the scaffold protein Apaf-1 assembles into the apoptosome—a caspase activation platform that subsequently cleaves and activates procaspase-9 to initiate apoptosis (Ruan et al., 2025).

Interestingly, while tumor cells evade intrinsic HOCl by expressing membrane-bound catalase, their extracellular superoxide anions readily react with exogenous HOCl. This reaction generates singlet oxygen, which subsequently inactivates tumor-surface superoxide dismutase (SOD). This inactivation creates a positive feedback loop, enhancing HOCl-mediated apoptosis by further increasing free superoxide levels (Bauer, 2013).

HOCl-modified macromolecules further amplify these apoptotic cascades. For instance, HOCl-altered mouse serum albumin (200 mg/L HOCl-MSA) facilitates Apaf-1-dependent caspase-9 activation (Wang et al., 2019), whereas HOCl-oxidized LDL (HOCl-LDL) induces apoptosis in acute T-cell leukemia via caspase-3 activation, PARP cleavage, and ROS accumulation (Resch et al., 2011). Similarly, hypochlorite-modified sphingomyelin (HOCl-SM) dissipates mitochondrial membrane potential, activates caspase-3, and causes DNA damage in dopaminergic PC12 cells (Nusshold et al., 2010). Furthermore, 2-Chlorohexadecanal (2-ClHDA)—a byproduct of HOCl-oxidized ether-phospholipids—compromises endothelial barrier function, disables mitochondria, and promotes ROS/caspase-3-driven apoptosis (Ullen et al., 2012).

Depending on the cellular context, HOCl can also exert anti-apoptotic effects. It can suppress apoptosis by directly oxidizing caspase-3 or indirectly inactivating it through hemoglobin destruction and free iron release (Jeelani et al., 2020). Additionally, HOCl-modified plasma proteins exhibit chaperone-like activity, preventing protein aggregation and enhancing neutrophil survival (Ulfig et al., 2019).

### 4.2 HOCl modulates cell proliferation

Hypoxia synergizes with HOCl to upregulate NF-κB expression in primary rat pulmonary artery smooth muscle cells (PASMCs). This activation enhances PASMC proliferation, apoptosis resistance, and migration—ultimately driving vascular remodeling and accelerating pulmonary hypertension (You et al., 2018). In macrophages, oxidized low-density lipoprotein (oxLDL) stimulates proliferation and accelerates atherosclerotic lesion progression (Zhang et al., 2024). Similarly, HOCl-mediated oxidation of the extracellular matrix (ECM) produced by human coronary artery smooth muscle cells (HCASMCs) reduces cell-matrix adhesion while stimulating proliferation, collectively facilitating plaque formation and atherosclerosis development (Cai et al., 2019).

### 4.3 HOCl accelerates cellular senescence

**FIGURE 3:**
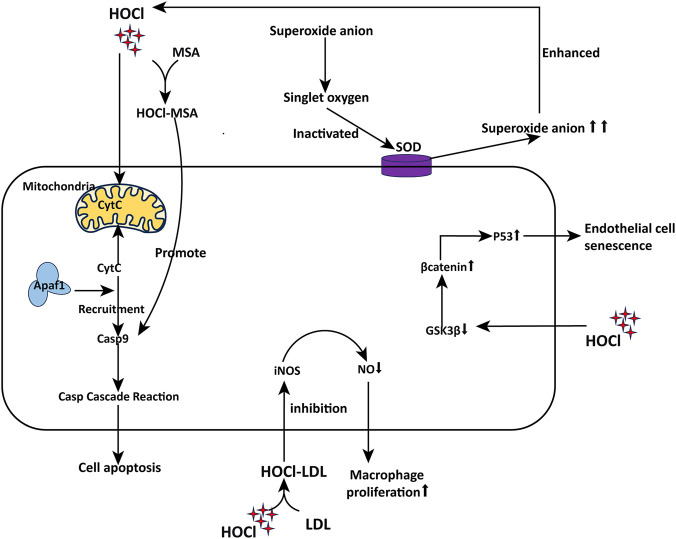
Regulation of Cell Fate by Hypochlorous Acid. HOCl regulates cell fate (apoptosis, proliferation, senescence, differentiation) via multiple pathways: it induces apoptosis through SOD inactivation and the cytC/Apaf1 cascade, drives senescence via β-catenin/p53, and modulates proliferation/differentiation through HOCl-LDL.

The accumulation of ROS, including HOCl, induces severe cellular DNA damage and functional impairment, thereby accelerating cellular senescence (Wen et al., 2024). In hyperlipidemic rat models, upregulated endothelial myeloperoxidase (MPO) expression enhances HOCl production, driving vascular endothelial cell senescence via the β-catenin/p53 pathway (Liu et al., 2024). Additionally, elevated concentrations of hydrogen peroxide (H_2_O_2_) directly accelerate oocyte aging and compromise their quality, a damaging process further exacerbated by H_2_O_2_-derived HOCl (Khan et al., 2015) (Figure 3).

### 4.4 HOCl drives pathological cell differentiation

Hypochlorous acid (HOCl) orchestrates pathological cell differentiation across multiple systems. For instance, the HOCl-activated probe CPP targets prolyl hydroxylase 2 (PHD2) at Cys302, quenching HOCl while inhibiting PHD2 activity. This stabilizes hypoxia-inducible factor-1α (HIF-1α), upregulates HEY1, and drives the transdifferentiation of human dermal fibroblast into vascular endothelial cells (Cui et al., 2022). Concurrently, HOCl-oxLDL accelerates monocyte-to-macrophage differentiation—evidenced by enhanced macrophage marker expression and diminished monocytic traits—promoting foam-cell formation and early atherosclerosis (Radhika and Sudhakaran, 2013). Within the vasculature, vascular peroxidase 1 (VPO1) generates HOCl to catalyze lipid peroxidation; the resultant HOCl-oxLDL induces osteoblastic differentiation of vascular smooth muscle cells, facilitating vascular calcification (Tang et al., 2015). Furthermore, advanced oxidation protein products (AOPPs) trigger a macrophage-to-dendritic-cell morphological transition. This AOPP-driven phenotypic shift likely accelerates atherosclerosis in chronic kidney disease (CKD) patients due to impaired AOPP clearance (Garibaldi et al., 2017).

### 4.5 HOCl-mediated disruption of organelle dynamics

Mitochondria, essential for cellular energy production, represent prime targets for HOCl-mediated oxidative damage (Cheshchevik et al., 2021). Although mitochondria are major sites of free radical generation, excessive HOCl accumulation severely compromises their integrity, inducing membrane permeabilization and ultimately cell death (Fan et al., 2025). This oxidative insult triggers the opening of the mitochondrial permeability transition pore (mPTP), releasing additional ROS into the cytosol. Depending on the severity of the oxidative stress, this mPTP disruption dictates distinct cell death pathways. Severe structural damage establishes a lethal “ROS-induced ROS release” positive-feedback loop, culminating in mitochondrial dysfunction and mPTP-dependent necrotic cell death (Robichaux et al., 2023). Conversely, specific modulation of mPTP dynamics and related signaling molecules by HOCl can directly promote apoptosis (Zhang et al., 2023). Interestingly, while conventional understanding restricted HOCl production to the MPO system of activated neutrophils, emerging research reveals that mitochondria themselves can endogenously generate HOCl under specific pathological conditions, particularly during oxidative stress (Mu et al., 2022). Indeed, recent studies have detected transient HOCl bursts during peripheral mitochondrial fission in COS-7 cells, suggesting its potential as both a diagnostic marker and a therapeutic target for mitochondrial-damage-related disorders (Zhang et al., 2023).

Beyond mitochondria, HOCl irreversibly disrupts endoplasmic reticulum (ER) calcium homeostasis, significantly exacerbating ER stress (Jiang et al., 2022). Crucially, the functional crosstalk between the ER and mitochondria plays an irreplaceable role in HOCl-induced cellular damage. HOCl triggers a massive release of calcium ions (Ca^2+^) from the ER into the cytosol (Cheshchevik et al., 2021). These Ca^2+^ ions are subsequently channeled into mitochondria via ryanodine receptors (RyRs) or inositol trisphosphate receptors (IP_3_Rs) located at ER-mitochondria contact sites. The resulting mitochondrial calcium overload forces the mPTP to open, collapses the mitochondrial membrane potential, and irreversibly initiates the apoptotic program (Cheshchevik et al., 2021).

Organelle-based signaling networks are also critical targets for exogenous environmental insults. For instance, oral ingestion of polystyrene microplastics, while not posing significant acute health risks to mammals, can induce ferroptosis and subsequent chronic liver damage (Stock et al., 2019). To monitor such organelle-specific stress, a research team developed TPAC-B, a fluorescent probe that specifically detects HOCl and targets dual organelles (mitochondria and lipid droplets), enabling the early diagnosis of polystyrene microplastic-induced, ferroptosis-associated liver injury (Cui et al., 2025). Similarly, another novel lipid droplet-targeting probe, NSSP, monitors dynamic changes in intracellular lipid droplets—specifically HOCl levels and polarity—highlighting its potential as a tool for the early diagnosis of NAFLD and cancer (Yang et al., 2025).

**FIGURE 4:**
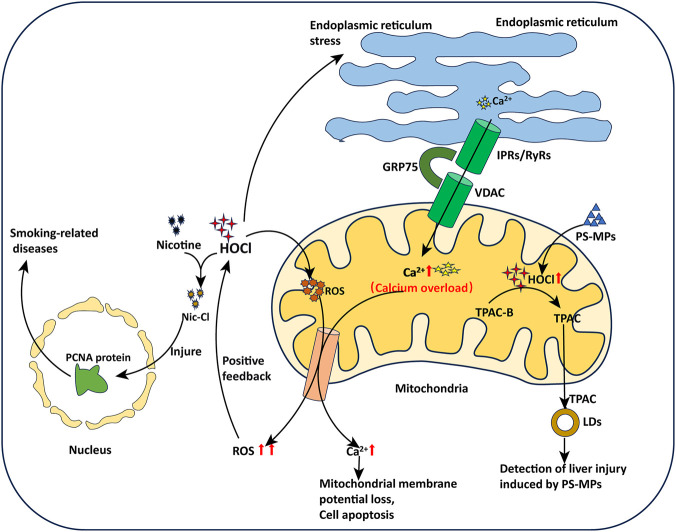
Relationship between HOCl and organelles. HOCl disrupts ER calcium homeostasis, causing mitochondrial calcium overload, ROS positive feedback and cell apoptosis; it forms Nic-Cl with nicotine to damage nuclear PCNA, and TPAC-B detects HOCl for PS-MPs-induced liver injury diagnosis.

Finally, HOCl exerts highly specific damaging effects within the nucleus. HOCl derived from infiltrating neutrophils reacts with nicotine from tobacco or e-cigarette smoke to generate nicotinic chloride (Nic-Cl). This compound causes dose-dependent damage to proliferating cell nuclear antigen (PCNA)—a key nuclear protein—inducing molecular lesions in the nuclei of intact cells. Such Nic-Cl-mediated protein damage may contribute to smoking-related pathologies (Salama et al., 2014) (Figure 4).

## 5 Dual roles of hypochlorous acid in tumor progression

The regulation of cell apoptosis and proliferation by HOCl translates into specific pathophysiological effects within the complex tumor microenvironment (Bauer, 2013; Valadez-Cosmes et al., 2023). By reshaping the local immune microenvironment, HOCl profoundly influences tumorigenesis, progression, and metastasis (Liu T. W. et al., 2021; Zhou et al., 2022; Lin et al., 2024).

Tumor development progresses through three stages: non-transformed cells, transformed cells (early malignancy), and true tumor cells (advanced disease). Critically, unlike non-transformed cells, transformed cells generate extracellular superoxide anion (O_2_•^-^) and subsequent HOCl (Bauer, 2013), positioning HOCl-sensitive probes as promising tools for early cancer detection. As previously discussed, HOCl can induce cell death. While prior research has established that cancer results from the accumulation of genomic mutations, recent studies highlight that sublethal cell death signaling—particularly necroptosis—plays a crucial role in initiating these mutations, thereby significantly contributing to tumorigenesis (Vucur et al., 2023).

### 5.1 Pro-tumorigenic mechanisms of HOCl

Extensive studies demonstrate that MPO-derived HOCl promotes malignancy through diverse pathways depending on the cellular context. Initially, HOCl induces progressive DNA damage by breaking hydrogen bonds, resulting in DNA double-strand dissociation. Concurrently, HOCl modifies the heterocyclic NH groups of guanine and thymine to form chloramines, which further exacerbates DNA double-strand dissociation and drives malignant transformation (Lin et al., 2024). Beyond genomic instability, HOCl promotes the conversion of proMMP9 into active MMP9, which accelerates extracellular matrix (ECM) degradation, enhances cell adhesion and proliferation, and ultimately drives cancer metastasis and disease progression (Wang et al., 2022). Furthermore, HOCl inhibits the activity of GRP78 by promoting oxidation at lysine 353, thereby suppressing autophagy and accelerating the growth of A549 lung cancer cells (Ning et al., 2019). Tissue-specific responses also exist; under normal circumstances, the TGF-β1/H_2_O_2_/HOCl axis promotes cellular senescence through the Smad and p38 MAPK pathways. However, in hepatocellular carcinoma cells, this axis continuously activates SIRT6. The resulting SIRT6 overexpression not only bypasses the senescence response but also actively drives liver cancer progression (Feng et al., 2015).

### 5.2 Anti-tumorigenic mechanisms of HOCl

**FIGURE 5:**
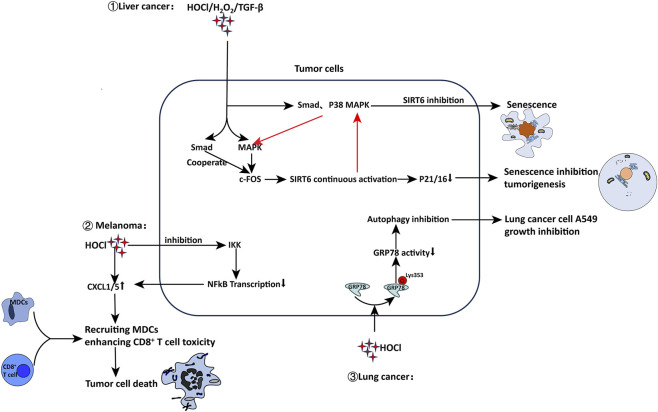
Roles of Hypochlorous Acid in Tumor Progression. HOCl exerts dual pro/anti-tumor effects: it activates SIRT6 (liver cancer) and oxidizes GRP78 (lung cancer) to promote tumorigenesis, and inhibits IKK/NF-κB in melanoma to recruit CD8^+^ T cells for tumor suppression.

Conversely, HOCl also exerts potent anti-tumor effects. Membrane-associated NADPH oxidase in tumor cells produces extracellular O_2_•^-^, which synergizes with the HOCl, NO, and peroxynitrite pathways. This synergy selectively induces apoptosis in transformed cells and inhibits tumor progression (Bauer, 2015). Furthermore, in early-stage melanoma, myeloid-derived cells (MDCs) possessing MPO activity produce HOCl, which acts as a trans-inhibitor of IKK, attenuating NF-κB transcriptional activation. This reduction in NF-κB signaling, potentially combined with the direct effects of HOCl itself, elevates plasma concentrations of the chemotactic cytokines CXCL1 and CXCL5. Consequently, this cytokine surge enhances the recruitment of CD8^+^ cytotoxic T cells to the tumor site, ultimately inhibiting melanoma growth and establishing an early anti-tumor defense (Liu T. W. et al., 2021) (Figure 5).

## 6 HOCl modulates tumor immunity and NET formation

**FIGURE 6:**
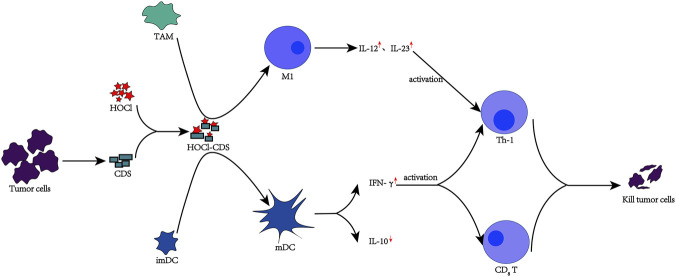
HOCl affects tumor cell immunity. HOCl-treated tumor CDS promotes DC maturation and TAM M1 polarization, modulates cytokine secretion (upregulates IL-12/IFN-γ, downregulates IL-10), and activates Th1/CD8^+^ T cells to exert anti-tumor effects.

Autologous tumor cells and their cell-derived secretions (CDS) possess the intrinsic capacity to induce anti-tumor immune responses. Notably, tumor CDS treated with hypochlorous acid (HOCl-CDS) significantly promotes dendritic cell (DC) maturation and drives tumor-associated macrophages (TAMs) toward M1 polarization, thereby exerting potent tumor-killing effects (Zhou et al., 2022). M1 macrophages exhibit a large, rounded morphology and orchestrate type I T cell responses through antigen presentation and the secretion of pro-inflammatory cytokines (e.g., IL-12 and IL-23), thereby inhibiting tumor growth (Boutilier and Elsawa, 2021; Wang Y. N. et al., 2023). Concurrently, HOCl-CDS reduces the levels of the DC-secreted anti-inflammatory cytokine IL-10 while elevating the pro-inflammatory cytokine IFN-γ, a profile crucial for the activation of CD8^+^ T cells and Th1 cells. This cytokine shift activates Th1 responses and initiates cytotoxic T lymphocyte (CTL) activation (Abbaspour et al., 2025) (Figure 6).

**FIGURE 7:**
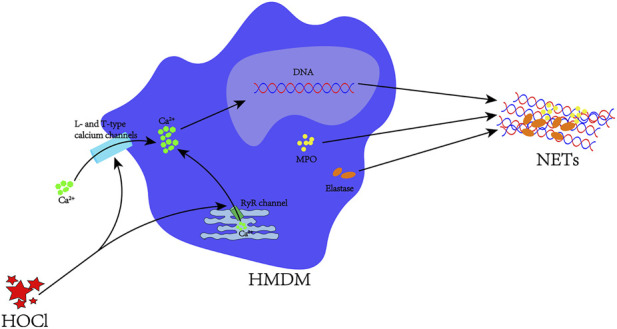
HOCl induces the generation of NETs. HOCl disrupts calcium homeostasis in HMDMs via L/T-type calcium channels and RyRs, promoting the release of DNA, MPO and elastase, which assemble extracellularly to form neutrophil extracellular traps (NETs).

The formation of neutrophil extracellular traps (NETs) is primarily driven by reactive oxygen intermediates (ROIs) generated through the catalytic action of NADPH oxidase. Within this process, HOCl serves as the key ROI that is both necessary and sufficient for inducing NET release (Palmer et al., 2012). Subsequent studies confirmed that when human monocyte-derived macrophages (HMDMs) are exposed to pathophysiological concentrations of HOCl, it severely disrupts cytoplasmic calcium homeostasis. Mechanistically, HOCl acts on L-type and T-type calcium channels on the plasma membrane and ryanodine receptors (RyRs) in the endoplasmic reticulum, subsequently promoting the dose-dependent release of DNA and histones into the extracellular space. These extracellularly released components intertwine to form a reticular structure comprising a DNA-chromatin scaffold decorated with functional proteins such as histones, myeloperoxidase (MPO), and elastase. This reticular structure constitutes the extracellular trap (NET) (Rayner et al., 2018). Furthermore, extracellularly generated NETs are closely associated with the onset and progression of various diseases, including COVID-19, sepsis, cancer, diabetes, and cardiovascular diseases (Jensen et al., 2023) (Figure 7).

Furthermore, HOCl-induced NETs exhibit distinct context-dependent roles in the tumor microenvironment. On the one hand, the DNA framework produced by NETs can trap circulating tumor cells (CTCs) and protect them from immune-mediated elimination, thus promoting tumor metastasis and development (Cools-Lartigue et al., 2013). Conversely, studies have also found that chemotherapy can recruit neutrophils and stimulate the formation of NETs to exert an anti-tumor function. Cathepsin G (CG) embedded within NETs enters tumor cells through the membrane receptor RAGE and promotes the translocation of BAX to mitochondria. This process triggers apoptosis and directly kills tumor cells, demonstrating potent anti-tumor efficacy (John Wang, 2026).

## 7 Clinical strategies targeting hypochlorous acid: therapeutics and diagnostics

As established, excessive hypochlorous acid (HOCl) production contributes to multiple pathologies—including atherosclerosis, rheumatoid arthritis, and cancer—thereby positioning HOCl as both a valuable diagnostic biomarker and a specific trigger for targeted drug delivery (Wei et al., 2019). Recently, researchers developed BClO, currently the most sensitive HOCl-responsive probe. In in vitro studies evaluating 6 cell lines (cancerous: HeLa, HepG-2, MCF-7; non-cancerous: COS-7, HL-7702, RWPE-1), BClO exhibited robust fluorescence in malignant cells but negligible signals in normal lines. This cancer-specific response demonstrates BClO’s potential as a highly specific tool for early tumor detection (Zhu et al., 2018).

Given that tumor cells exhibit heightened sensitivity to redox homeostasis imbalances (Glorieux et al., 2024), HOCl enrichment in solid tumors serves as a key trigger for selective therapies. As detailed in Table 1, various HOCl-activated prodrugs, self-assembling nanocarriers, and responsive sensors (e.g., DHU-CBA2, Au-MB-PEG, ZBM-H) have been developed to target tumor proliferation and impede metastasis (Liu D. et al., 2021; Ning et al., 2022; Wang et al., 2024). Beyond molecular targeting, HOCl effectively potentiates immunotherapy. Several HOCl-modified tumor cell vaccines and autologous dendritic-cell preparations have demonstrated robust immune activation in both murine models and early-phase clinical trials (Table 1) (Zhou et al., 2012; Graciotti et al., 2020). For peritoneal malignancies, HOCl demonstrates superior cytotoxicity compared to other reactive species, suggesting potential synergy with hyperthermic intraperitoneal chemotherapy (HIPEC) (Freund et al., 2021). Mechanistically, this superior potency is attributed to HOCl’s exceptionally rapid reaction kinetics with sulfur-containing amino acids and intracellular antioxidants. Unlike hydrogen peroxide, HOCl induces rapid and irreversible protein damage and severely depletes glutathione reserves before tumor cells can mount an adaptive antioxidant response (Freund et al., 2021; Andrés C. M. C. et al., 2022).

**TABLE 1:** Summary of translational maturity and developmental stages of HOCl-targeted clinical strategies.

Agent/Strategy	Function/Target	Target disease/Model	Development stage	References
BClO probe	Ultrasensitive HOCl-responsive fluorescent probe	Solid tumors (HeLa, HepG-2, MCF-7)	Preclinical (In vitro)	Zhu et al. (2018)
DHU-CBA2	HOCl-activated COX-2 inhibitor	Lung cancer and spinal metastases	Preclinical (In vivo)	Wang et al. (2024)
HOCl-oxidized CT26 cell vaccine	Elicits humoral/cellular tumor immunity	Colorectal cancer	Preclinical (In vivo)	Zhou et al. (2012)
HOCl-producing e-bandages	Eradication of *P. aeruginosa* biofilms	Wound infections	Preclinical (In vivo)	Fleming et al. (2023)
Autologous DC vaccine (HOCl-treated lysates)	Induces multifunctional CD4^+^ T-cell responses	Ovarian cancer	Phase I clinical trial	Graciotti et al. (2020)
Adjunctive HOCl wound solution	Accelerates healing of chronic wounds	Diabetic foot and venous leg ulcers	Clinical application	Jull et al. (2021)
HOCl eye drops	Promotes healing with minimal adverse events	Fungal keratitis	Clinical application	Wang et al. (2023a)
Stabilized HOCl core formulations	Sustained delivery with regulatory approval	Dermatology and wound care	Clinical application	Del Rosso and Bhatia (2018)

Conversely, in systemic inflammatory conditions like sepsis, intravenous high-dose vitamin C acts as a potent MPO inhibitor to reduce HOCl generation and improve survival outcomes (Wen et al., 2023; Peskin et al., 2025). Furthermore, in the context of oncology, pharmacological high-dose vitamin C acts as a pro-oxidant, selectively generating cytotoxic levels of H_2_O_2_ in the tumor microenvironment via electron transfer to catalytic metal ions (Ali et al., 2024), which complements its modulation of the MPO-HOCl axis. Furthermore, topical HOCl exhibits multifaceted therapeutic efficacy across dermatology, ophthalmology, and wound management (Table 1). Recent advancements in stabilized core formulations and innovative delivery systems—such as electrochemical “e-bandages” and specialized hydrogels—have overcome HOCl’s inherent instability and significantly expanded its clinical utility in treating infections, radiodermatitis, and skin malignancies (Leung et al., 2013; Del Rosso and Bhatia, 2018; Jandova et al., 2021; Jull et al., 2021; Zhou et al., 2022; Fleming et al., 2023; Wang H. et al., 2023).

Despite progress in related research, achieving precise detection of endogenous hypochlorous acid (HOCl) within the complex tumor microenvironment remains fraught with significant technical bottlenecks. First, the inherent chemical instability and extreme reactivity of HOCl make direct, accurate *in vivo* quantification exceptionally difficult (Jeelani et al., 2020). Because HOCl reacts almost instantaneously with nearby biological macromolecules, its biological half-life is exceedingly short (Winterbourn, 2008). Furthermore, the vast majority of current HOCl fluorescent probes are reaction-based and therefore irreversible (Wu et al., 2019). While they can detect the cumulative level of HOCl, they fundamentally fail to capture the real-time, dynamic physiological fluctuations—both the generation and clearance—of this oxidant. Second, maintaining absolute selectivity remains a persistent challenge. The tumor microenvironment is characterized by severe oxidative stress, simultaneously generating multiple reactive oxygen and nitrogen species (ROS/RNS) such as hydrogen peroxide and peroxynitrite. Designing probes that can exclusively distinguish HOCl without false-positive interference from these highly similar competing oxidants requires complex molecular engineering (Zhao et al., 2025).

## 8 Summary and conclusions

Hypochlorous acid (HOCl)—a pivotal myeloperoxidase-derived reactive oxygen species—exerts complex dual roles in diverse pathologies, particularly cancer. This review synthesizes the mechanisms of HOCl generation, biomolecular modifications (notably protein chlorination/oxidation), cell fate regulation (apoptosis, proliferation, senescence, differentiation), and organelle interactions. In oncology, HOCl acts as a double-edged sword: pro-tumor effects include promoting survival via caspase-3 inactivation and GRP78 oxidation and driving metastasis through MMP activation; meanwhile, anti-tumor effects involve selective apoptosis induction by exploiting redox imbalance and extracellular superoxide anion pathways. Emerging strategies leverage HOCl’s dual identity as a biomarker and pathophysiological driver. While some approaches, such as novel topical formulations and specific immunotherapies, have advanced to clinical application or Phase I trials, many targeted modalities remain in preclinical development (Table 1). Currently, ultrasensitive probes enable early cancer detection in laboratory settings; HOCl-activated prodrugs/drug-delivery systems target tumor microenvironments in murine models; immunotherapies utilize HOCl-modified tumor cells/lysates (Phase I trials); and novel stabilized topical formulations show efficacy in wound healing (clinical application), radiodermatitis, and skin cancer chemoprevention.

## 9 Future perspectives

Currently available tools often exhibit critical optical and spatial limitations (Mu et al., 2022). Many conventional probes operate in the visible light spectrum, suffering from severe scattering and auto-fluorescence in dense tumor tissues, underscoring the urgent need for advanced near-infrared (NIR) probes with large Stokes shifts. Additionally, given that HOCl triggers distinct cell fate responses depending on its specific origin—such as promoting apoptosis via mitochondria (Neto et al., 2026) or mediating immune defense in lysosomes (Thekkan et al., 2019)—probes lacking robust organelle-targeting capabilities cannot accurately map its dynamic spatial distribution *in vivo*. Overcoming these specific bottlenecks regarding reversibility, selectivity, and spatiotemporal resolution will be critical for the next-generation of HOCl-targeted translational research. Moreover, future research should explore rational combination strategies that pair H_2_O_2_-generating agents (such as pharmacological ascorbate or metal-based prodrugs) with HOCl-activated targeted therapeutics, potentially overcoming tumor heterogeneity and synergistically amplifying ROS-mediated cancer cell death (Ali et al., 2024).
